# Staged, hybrid approach by zone 2 arch replacement and completion thoracic endoprosthesis in retrograde acute type A aortic dissection

**DOI:** 10.1016/j.jvscit.2024.101663

**Published:** 2024-10-25

**Authors:** Nabil Saouti, Guillaume S.C. Geuzebroek, Sjoerd F.M. Jenniskens, Robin H. Heijmen

**Affiliations:** a Department of Cardio-Thoracic Surgery, Radboud Umc, Nijmegen, The Netherlands; b Department of Radiology, Radboud Umc, Nijmegen, The Netherlands

**Keywords:** Retrograde type A dissection, Zone 2 arch replacement, Staged hybrid treatment, TEVAR, Remodeling

## Abstract

We describe a case of retrograde acute type A aortic dissection approached by a hybrid, staged approach consisting of a zone 2 arch replacement and completion thoracic endovascular aortic repair procedure combined with distal balloon-assisted stent graft dilatation to prevent retrograde false lumen flow. This technique may be an alternative and more complete when compared with a frozen elephant trunk procedure at onset. Additionally, favorable remodeling of the entire thoracic aorta is observed.

Retrograde acute type A aortic dissection (RTAAD) is defined as a proximal extension of the false lumen (FL) toward the ascending aorta, originating from a downstream dissection with the primary intimal entry tear distal to the left subclavian artery (LSA). Given the risks of the proximal intrapericardial aortic dissection urgent repair is indicated. When and how to deal with the (distal) primary entry tear is still debated on.^1^, ^2^, ^3^ The patient agreed to the publication of his case details and images.

## Case report

**Fig 1:**
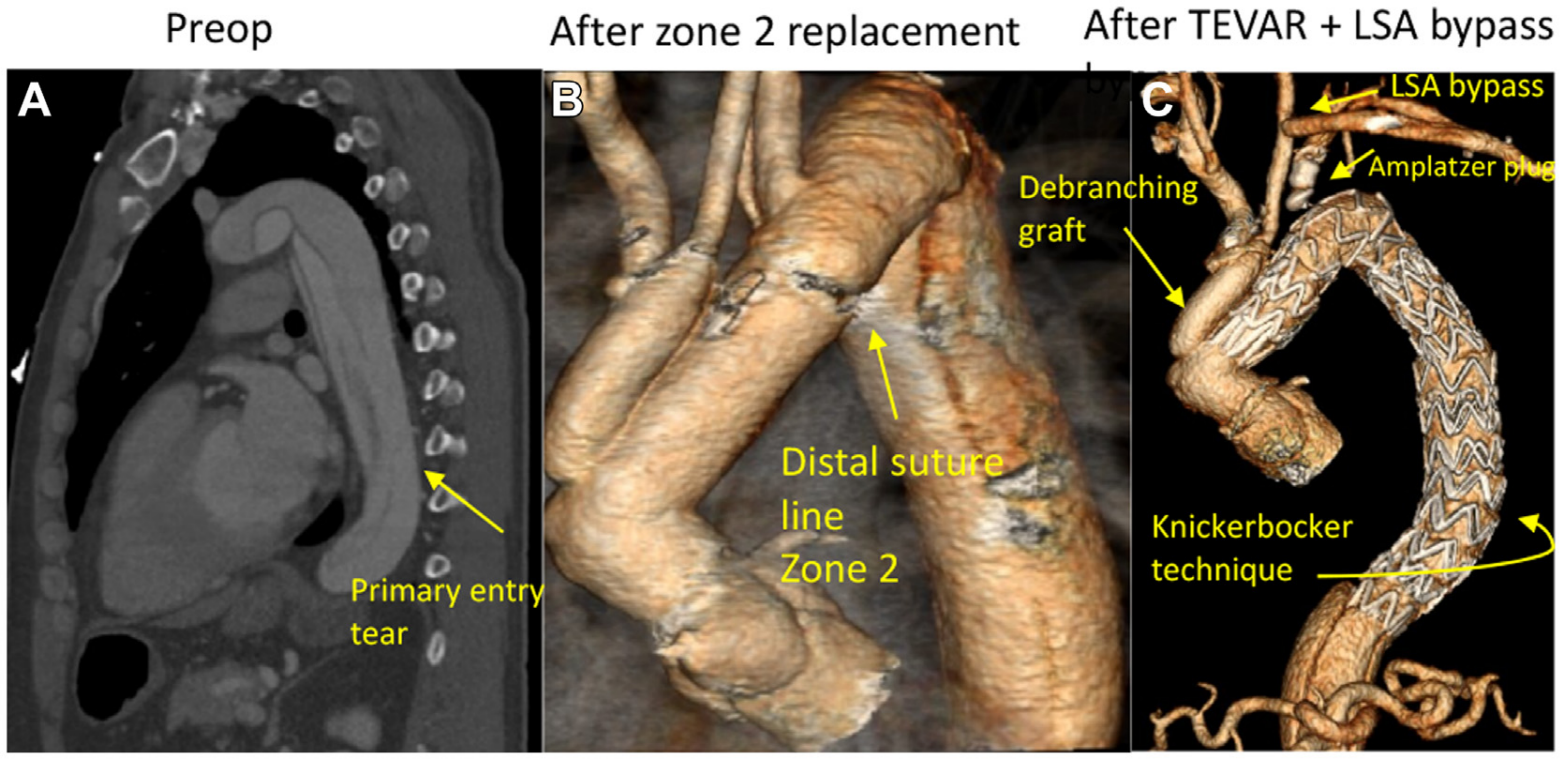
**(A)** Sagittal view computed tomography (CT) scan of retrograde type A dissection with the primary entry in the descending thoracic artery (arrow). **(B)** CT reconstruction after zone 2 arch replacement showing distal suture line just proximal to left subclavian artery (LSA). **(C)** CT reconstruction after staged thoracic endovascular aortic repair (TEVAR) starting just distal to the 16 mm debranching graft in the proximal ascending aorta to the celiac trunk, level of the Knickerbocker technique is depicted. Note also the LSA-left common carotid artery (LCCA) bypass and the Amplatzer plug in the proximal LSA (to prevent endoleak).

In this case report, we describe a 53-year-old male patient presenting with acute, typical chest pain. He was hemodynamically stable, neurologically without any deficits. An electrocardiograph-triggered computed tomography (CT) scan showed a dissected aorta that extended from the root to both iliac arteries. The intimal lamella was completely intact proximally. The primary, proximal entry tear appeared to be located in the proximal descending aorta, about 10 cm distal to the LSA. Distally, the true lumen (TL) was narrowed but patent, without signs of malperfusion (Fig 1). We, therefore, diagnosed a RTAAD (or according to the recent SVS/STS reporting standards an acute type B_0,10_).^4^

Given the intrapericardially dissected ascending aorta with a patent FL, emergent intervention was indicated. The presence of a patent FL proximally (ie, no proximal landing zone), excluded an endovascular repair by stent grafting (thoracic endovascular aortic repair [TEVAR]) at the level of the descending aorta to seal the intimal entry. Therefore, we opted for emergent open surgery by median sternotomy and cardiopulmonary bypass with femoral arterial cannulation.

Intraoperatively, indeed no intimal tear was visible in the ascending aorta, confirming the diagnosed RTAAD. We performed a supracoronary ascending aorta replacement with a so-called zone 2 aortic arch resection using bilateral antegrade selective cerebral perfusion at 25°C core temperature. A left vertebral artery (LVA) that originated directly from the aortic arch, which was slightly larger in size than the right vertebral artery, had adequate backflow and was left undisturbed because the distal suture line could be made just proximal to the LVA between the left common carotid artery (LCCA) and LSA (ie, zone 2). Suture lines were reinforced using the Teflon felt neomedia technique and an extra circular Teflon felt on the adventitial layer. A separate 16-mm vascular prothesis was used to debranch the brachiocephalic trunk (BCT) and the LCCA using the island technique, to a more proximal location at the ascending aorta graft thereby creating a future landing zone for TEVAR of approximately 3 cm in length (Fig 1), as described previously by us and others.^5^, ^6^, ^7^ The postoperative course was uneventful. Noteworthy, pathological examination confirmed that there was no intimal disruption in the resected aorta and no evidence for connective tissue disease.

Before discharge, 6 days after the index operation, a control CT scan showed an increase in total aortic diameter (TL and FL) of the proximal descending thoracic aorta (DTA) from 40 mm to 43 mm. To prevent further dilatation and, hence, the development of a postdissection aneurysm the case was accepted for completion TEVAR in the (ideal) subacute phase, preemptively stent grafting from zone 2 to the lower DTA to exclude the FL and induce thrombosis and subsequent favorable remodeling. Given the LVA from the arch, an additional CT scan of the circle of Willis demonstrated adequate collateral circulation to the brain allowing acute occlusion of the LVA in case of TEVAR.

A total of 6 weeks after the index surgery, the patient was readmitted electively for completion TEVAR, with concomitant LSA-LCCA bypass grafting. Because the LSA was dissected proximally, we used an infraclavicular access to the LSA (with an 8-mm Dacron prosthesis).^8^^,^^9^ The LVA was clipped surgically and the LSA was plugged proximally endovascularly to prevent retrograde endoleaks, followed by a percutaneous TEVAR procedure using 3 Relay PRO stent grafts (34 mm, Terumo Aortic, Bolton Medical, Sunrise, FL), starting from zone 2 to just proximal to the celiac trunk. Two stentrings from its distal end, the distal stent graft was balloon-dilated (Reliant, Medtronic Inc, Minneapolis, MN), the so-called Knickerbocker technique as described in detail previously.^10^^,^^11^ Completion fluoroscopy showed no endoleaks, a completely excluded FL at the entire thoracic level, as well as a patent LCCA-LSA bypass graft (Fig 1). The patient was extubated in the hybrid room, neurologically intact, and had an uneventful recovery.

**Fig 2:**
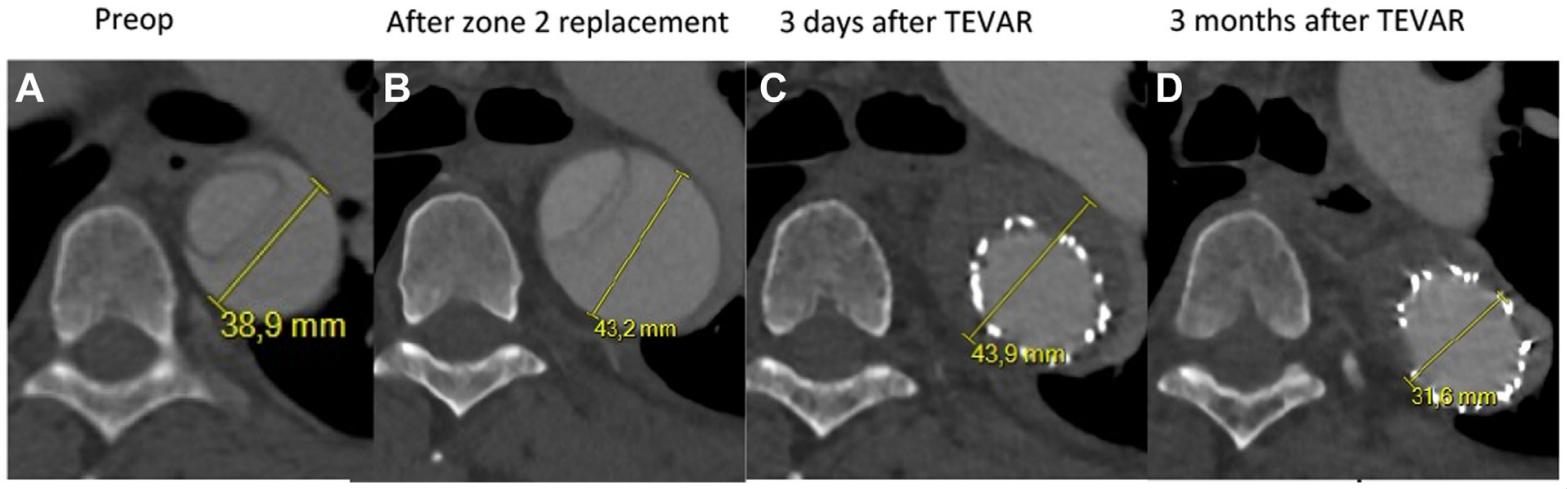
Coronal view of the computed tomography (CT) scans all at the same level (trachea bifurcation) showing dissection with total diameter preoperatively **(A)** and postdissection dilatation **(B)**, result after thoracic endovascular aortic repair (TEVAR) **(C)** and favorable remodeling 3 months after TEVAR **(D)**.

Before discharge, a control CT scan showed a completely thrombosed FL at the entire length of the DTA in continuation with the earlier zone 2 aortic arch repair. At the abdominal level, FL perfusion persisted as expected. At the 3-month follow-up, the entire DTA had remodeled completely (Fig 2).

## Discussion

In 7% to 25% of patients with acute aortic type A dissection, the primary entry tear occurs in the DTA.^12^, ^13^, ^14^ To resect the primary entry tear distal to the LSA in addition to the required ascending aortic repair requires extensive aortic arch surgery, which is complex technically (especially in the acute setting), with longer circulatory arrest times and the associated higher risks of morbidity and mortality. The primary entry tear may even be out of surgical reach using a midsternal approach. The frozen elephant trunk (FET) technique (using a hybrid graft) has been used as an alternative, offering a single-staged solution for RTAAD. By covering the downstream primary entry tear using a stent graft distally, the FET has the potential to resolve distal malperfusion by expanding the TL. However, this technique requires surgical experience, and may be considered too complex, especially in the acute setting, and hazardous (given the associated risk of spinal cord ischemia) to be used routinely.^2^ Additionally, the current commercially available hybrid prostheses may not be ideal in the acutely dissected aorta with a fragile intimal membrane, given the relatively high rate of distal stent-induced new entries.^15^

Instead of a single-staged approach to treat our patient with RTAAD at the index procedure using the FET, which we would have preferred in case of distal malperfusion (as also recommended in the guideline),^2^ we opted for a staged, zone 2 arch repair with completion TEVAR upon indication at a later stage. We have now adopted successfully this approach at our institution for already some years for the majority of our acute TAAD patients (DeBakey type I) with a sufficient expected longevity (arbitrarily for patients <70 years of age).^5^, ^6^, ^7^

When compared with the FET technique in the acute setting, our staged approach is technically simpler (requiring a circular, end-to-end anastomosis at zone 2) and, most important, does not increase the risk of spinal cord ischemia, but still leaves the option for endovascular completion. Subsequent, staged TEVAR can be individualized with respect to indication (predictors of poor outcome, ie, progressive postdissection dilatation), timing (subacute phase, lower risk but still remodeling), extent (complete DTA), and type of stent graft (lower risk of stent-induced new entries), as nicely shown in the current case.

Because the entire DTA is stented to the celiac trunk, at the second stage we revascularized the LSA with a bypass to decrease the risk of spinal cord ischemia. Only if the patient is missing abdominal lumbar arteries and/or iliac internal arteries would preemptive liquor drainage have been applied. We favor LSA-LCCA bypass as an alternative to a thoracic branch endoprosthesis^6^ because it is much cheaper and, therefore, more widely applicable. In contrast with the study by Desai et al,^6^ we prefer to extend the TEVAR from zone 2 to the celiac trunk in the subacute phase (ie, window of plasticity) in combination with a modified Knickerbocker technique^11^ to exclude retrograde FL flow and induce thrombosis and positive remodeling along the entire thoracic aorta. In contrast with these previous studies,^6^^,^^7^ we prefer one common trunk (ie, a 16-mm graft) to both the arch branches (ie, BCT and LCCA) as an island because it gives slightly more proximal landing zone for later TEVAR; especially in cases with dissection, not separating the arch branches is easier and carries less of a risk of disintegrating the arch vessels. Additionally, the island is kept small and only incorporates aortic tissue that is located in between the BCT and LCCA.

Given the absence of distal malperfusion, no provisional extension to induce complete attachment technique was added distally, because that would otherwise cover the visceral ostia, complicating open repair in case of late aneurysmal formation abdominally.

In light of the very recent guidelines for acute type A dissection that endorse emergent FET in the presence of a primary entry tear in the outer curve of the arch or within 10 cm distal to the LSA,^16^ with the current case we would like to emphasize a simplified alternative of zone 2 arch replacement plus staged completion TEVAR, and demonstrate that, by adding an adjunct to prevent FL flow, positive remodeling of the downstream aorta along the TEVAR occurs offering potentially a complete treatment for the entire thoracic aorta.

## Disclosures

None.

## References

[1] Malaisrie S.C., Szeto W.Y., Halas M. (2021). 2021 the American Association for Thoracic Surgery expert consensus document: surgical treatment of acute type A aortic dissection. J Thorac Cardiovasc Surg.

[2] Czerny M., Schmidli J., Adler S. (2019). Current options and recommendations for the treatment of thoracic aortic pathologies involving the aortic arch: an expert consensus document of the European Association for Cardio-Thoracic Surgery (EACTS) and the European Society for Vascular Surgery (ESVS). Eur J Cardiothorac Surg.

[3] Shrestha M., Bachet J., Bavaria J. (2015). Current status and recommendations for use of the frozen elephant trunk technique: a position paper by the vascular domain of EACTS. Eur J Cardio Thorac Surg.

[4] Lombardi J.V., Hughes G.C., Appoo J.J. (2020). Society for Vascular Surgery (SVS) and Society of Thoracic Surgeons (STS) reporting standards for type B aortic dissections. Ann Thorac Surg.

[5] Smith T., Heijmen R.H. (2021). Staged, hybrid approach to acute DeBakey type I aortic dissection. J Vis Surg.

[6] Desai N.D., Hoedt A., Wang G. (2018). Simplifying aortic arch surgery: open zone 2 arch with single branched thoracic endovascular aortic repair completion. Ann Cardiothorac Surg.

[7] Glauber M., Murzi M., Farneti P. (2011). Aortic arch replacement with prophylactic aortic arch debranching during type A acute aortic dissection repair: initial experience with 23 patients. Eur J Cardio Thorac Surg.

[8] van der Weijde E., Saouti N., Vos J.A., Tromp S.C., Heijmen R.H. (2018). Surgical left subclavian artery revascularization for thoracic aortic stent grafting: a single-centre experience in 101 patients. Interact Cardiovasc Thorac Surg.

[9] Mandigers T., Smeenk H., Heijmen R. (2021). Surgical bypass from the left common carotid artery to the left subclavian artery: infraclavicular approach. Multimed Man Cardiothorac Surg.

[10] Kölbel T., Carpenter S.W., Lohrenz C., Tsilimparis N., Larena-Avellaneda A., Debus E.S. (2014). Addressing persistent false lumen flow in chronic aortic dissection: the knickerbocker technique. J Endovasc Ther.

[11] de Beaufort H.W.L., Vos J.A., Heijmen R.H. (2022). Initial single-center experience with the knickerbocker technique during thoracic endovascular aortic repair to block retrograde false lumen flow in patients with type B aortic dissection. J Endovasc Ther.

[12] Lansman S.L., Raissi S., Arisan Ergin M., Griepp R.B., York N. (1989). Urgent operation for acute transverse aortic arch dissection. J Thorac Cardiovasc Surg.

[13] Kazui T., Tamiya Y., Tanaka T., Komatsu S. (1996). Extended aortic replacement for acute type A dissection with the tear in the descending aorta. J Thorac Cardiovasc Surg.

[14] Kaji S., Akasaka T., Katayama M. (2003). Prognosis of retrograde dissection from the descending to the ascending aorta. Circulation.

[15] Kreibich M., Bünte D., Berger T. (2020). Distal stent graft–induced new entries after the frozen elephant trunk procedure. Ann Thorac Surg.

[16] Czerny M., Grabenwöger M., Berger T. (2024). EACTS/STS guidelines for diagnosing and treating acute and chronic syndromes of the aortic organ. Eur J Cardio Thorac Surg.

